# A latent profile analysis of cognitive emotion regulation strategies in relation to negative emotions and NSSI among Chinese junior high school students

**DOI:** 10.1186/s13034-024-00838-5

**Published:** 2024-12-04

**Authors:** Peiyu Zhang, Yuanqi Xiong, Jingyu Shi

**Affiliations:** 1 Tongji University School of Medicine, No. 500 Zhennan Road, Putuo District, Shanghai, 200331 China; 2 Clinical Research Center for Mental Disorders, Shanghai Pudong New Area Mental Health Center, Tongji University School of Medicine, Shanghai, 200124 China; 3 Department of Medical Humanities and Behavioral Sciences, School of Public Health, Tongji University School of Medicine, No. 500 Zhennan Road, Putuo District, Shanghai, 200331 China; 4 School of Haiyuan, Kunming Medical University, Kunming, 650106 Yunnan Province China

**Keywords:** Latent profile analysis, Cognitive emotion regulation, Negative emotions, NSSI, Chinese adolescents

## Abstract

**Background:**

Little is known about the latent profiles of cognitive emotion regulation strategy (CERS) and its relationship with negative emotions and Non-Suicidal Self-Injury (NSSI) in Chinese junior high school students, although CERS is thought to be strongly associated with emotional-behavioral problems in adolescents.

**Methods:**

A total of 2807 junior high school students in Yunnan Province, China, were selected for the study. They were measured with the Cognitive Emotion Regulation Questionnaire (CERQ), the Non-Suicidal Self-Injury Questionnaire, and the Depression-Anxiety-Stress Scale. Latent profile analysis was used to explore latent profiles of CERS among students, and the one-way ANOVA or *c*^2^ test was used to explore the relationship between the profiles and depression, anxiety, stress or NSSI.

**Results:**

(1) Latent profile analysis revealed five CERS types: ‘Maladaptive group’ (32.25%), ‘Moderate adaptive-low maladaptive group’ (24.68%), ‘Rigid group’ (19.73%), ‘High adaptive-moderate maladaptive group’ (14.42%), and ‘Sensitive group’ (8.82%). (2) The multivariable logistic regression analyses showed that with increasing age (*OR* = 0.812, 95% *CI* = 0.66–0.99, *p* < 0.05), junior high school students were less likely to be in the ‘Moderate adaptive-low maladaptive group’, and males (*OR* = 0. 698, 95% *CI* = 0.52–0.94, *p* < 0.05) were less likely to be classified as ‘Moderate adaptive-low maladaptive group’; (3) ANOVA and *c*^2^ test results showed that the differences between the different latent profiles of junior high school students on anxiety, depression, stress and NSSI indicators were statistically significant (*p* < 0.05). The ‘Sensitive group’ had the highest risk of emotional-behavioral problems and the ‘Moderate adaptive-low maladaptive group’ had the lowest risk of emotional-behavioral problems.

**Conclusion:**

Negative emotions and NSSI in junior high school students are closely related to their CERS profiles, and it is important to use targeted strategies to prevent and intervene in emotional-behavioral problems for individuals with different CERS subtypes.

**Supplementary Information:**

The online version contains supplementary material available at 10.1186/s13034-024-00838-5.

Adolescence is a crucial period of physical and mental development, as well as a high-risk period for mental disorders in lifespan. Junior high students are in the initial stages of adolescence, lacking stable emotional regulation and stress coping skills, which make them find it hard to deal with rapid internal and external changes, putting them at risk of mental health problems, especially for emotion problems and Non-Suicidal Self-Injury (NSSI) [1–3]. A meta-analysis revealed that the prevalence of anxiety, depression and self-injury among junior high school students in China over the past 10 years were 27%, 24% and 22%, respectively [4]. The high prevalence of negative emotional problems and NSSI not only has a serious impact on the health and social functioning of adolescents, but also leads to a burden of care and health economic costs which is a persistent obstacle to family and social development [5–7]. Thus, investigating the factors and mechanisms influencing negative emotions and NSSI among junior high school students is important.

Recent studies have confirmed that maladaptive cognitive activities and emotion regulation processes may be potential risk factors for the development and maintenance of anxiety, depression, and NSSI [8–11]. Cognitive-emotional model integrates the research on cognitive and emotional dysfunction inducing emotional-behavioral problems, and points out that impaired emotion regulation, especially the lack of adaptive cognitive emotion regulation strategies (CERS) leads to an increased risk of negative emotional responses and NSSI in stressful situations [12, 13]. CERS refer to conscious strategies used by individuals to adapt to stress and emotional distress, with the aim of regulating emotional experience and expression, including adaptive and maladaptive cognitive emotion regulation strategies [14]. Adaptive CERS refer to positive cognitive processes and behaviors, such as positive reappraisal, acceptance, refocusing on planning, positive refocusing, and putting into perspective, that individuals use to effectively manage their emotional responses to stressors and enhance resilience and well-being. Maladaptive CERS refer to negative cognitive processes and behaviors, such as rumination, self-blame, catastrophizing, and blaming others, that individuals use to cope with emotional distress, often resulting in exacerbated negative emotions and poor psychological outcomes. Individuals may experience different mental health outcomes as a result of choosing different CERS. Junior high school students are in early adolescence and have limited cognitive emotion regulation development. They tend to use less adaptive CERS and more maladaptive CERS, which correlates with higher levels of depression and anxiety [15]. Researches also noted that adaptive CERS significantly negatively predicted depression and NSSI among adolescents, while maladaptive CERS significantly positively predicted depression and NSSI [16, 17].

According to Self-Determination theory, the CERS used by an individual is not a specific strategy, but rather an integrative emotion-regulating process [18], which means individuals will inevitably use other CERS along with their tendency to adopt one CERS. Furthermore, there are significant individual differences in CERS [19]. Therefore, the use of adaptive or maladaptive tendencies to differentiate between complex CERS may be too simplistic and does not effectively reflect the simultaneous emergence of different modes of CERS. Latent Profile Analysis (LPA) is an individual-cantered approach to classify subjects based on the pattern of their responses, and to better analyze subgroup characteristics within each category [20]. By identifying the latent subgroups of CERS, researchers can go beyond the existing adaptive or maladaptive tendencies to explore more deeply the mixing patterns between CERS, and develop more efficient and targeted prevention and intervention for psychopathological problems among adolescents with different CERS subtypes.

In recent years, researchers from various countries have increasingly turned their attention to the utilization of LPA for investigating the association between heterogeneity within CERS and emotional-behavioral issues in adolescents. Heuvel (2020) et al. have identified four latent profiles of CERS in both non-clinical and clinically depressed populations in the Netherlands, namely ‘Low Regulators’ (10%), ‘High Regulators’ (44%), ‘Maladaptive Regulators’ (12%), and ‘Adaptive Regulators’ (34%). It was observed that individuals characterized as ‘Maladaptive Regulators’ exhibited higher levels of depressive symptoms [21]. Similarly, Chen (2018) et al. found three latent profiles of CERS (‘Low reaction’ (13%), ‘Medium reaction’ (68%), and ‘High reaction’ (19%)) among students from Chinese secondary schools, with those classified as ‘High reaction’ showing a high prevalence of unprotected sex behaviors [22]. These studies not only highlight the internal heterogeneity within CERS but also demonstrate variations in depressive symptoms and unprotected sex behaviors across different CERS profiles, which provides a new perspective on the important role CERS plays in causing emotional and behavioral problems among adolescents. Nevertheless, there is no study to explore the relationship between latent profiles of CERS and negative emotions and NSSI.

In order to explore the internal heterogeneity of CERS in Chinese adolescents and analyze the association of latent CERS profiles with negative emotions and NSSI, the present study used LPA to explore the sub-types of CERS among Chinese junior high school students, and further compared the differences in anxiety, depression, stressful emotions, and NSSI among individuals with different latent profiles of CERS. The aim was to provide a reference for accurate prevention and intervention of negative emotions and NSSI among Chinese junior high school students.

## Method

### Participants

A total of 2807 students from two junior high schools in Yunnan Province, China participated in and completed the questionnaire survey online. After excluding invalid questionnaires, we collected data from 2711 junior high students (50.50% were males and 49.50% were females), with a mean age of 12.89 (*SD* = 0.73). And 369 individuals were only children (13.61%) and 551 individuals had left behind experience (20.32%).

Before the survey, all participants and their parents signed the digital informed consent form indicating their willingness to participate in the study. After reading the study instructions, participants completed the online questionnaire by clicking on the link in the computer classrooms. After the survey, all participants could join one session of psychosocial support group for free if they need for compensation. All study procedures were approved by the Ethical Review Board of Kunming Medical University, School of Haiyuan (No. KMMUHYC2024MEC00005).

### Measures

#### Socio‑ demographic questionnaire

The self-designed questionnaire (Table S1) developed for current study was used to obtain socio-demographic variables including age, gender, left behind experience, only children or not.

#### Cognitive emotion regulation questionnaire (CERQ)

The Chinese version of CERQ developed by Zhu et al. [23] was applied to assess the CERS among junior high students. It is a 36-item self-report questionnaire, adopting 5-point Likert scale (from 1 = never to 5 = always). There are nine dimensions in the questionnaire: Acceptance, Positive refocusing, Refocus on planning, Positive reappraisal, Putting into perspective, Self-blame, Rumination, Catastrophizing, and Blaming others. Each dimension represented a specific type of CERS, and the dimension score was calculated by summing the item scores and dividing by the number of items in that dimension. Scores for adaptive CERS are the sum of scores for the initial five dimensions, while scores for maladaptive CERS are the sum of scores for the remaining four dimensions, with higher scores suggesting a higher frequency of using one specific CERS. The reliability and validity of the Chinese version of CERQ among Chinese adolescent were satisfactory [17]. In current study, the Cronbach’s α is 0.93. And the Cronbach‘s α of each subscale of CERS is detailed in Table 1.

**Table 1:** Mean and standard deviation of CERQ (*n* = 2711)

CERQ	***M***	***SD***	Cronbach’s α
Acceptance	3.18	1.06	0.81
Positive refocusing	2.86	1.02	0.81
Refocus on planning	3.39	1.11	0.91
Positive reappraisal	3.29	1.10	0.91
Putting into perspective	2.39	0.95	0.74
Self-blame	2.63	0.89	0.75
Rumination	2.80	0.99	0.77
Catastrophizing	2.02	0.97	0.85
Blaming others	1.91	0.82	0.81

#### Non-suicidal self-injury questionnaire (NSSI)

A questionnaire developed by You et al. [24] was conducted to measure whether seven common Non-Suicidal Self-Injurious behaviors occurred in individuals over the past six months. Each item is rated on a 4-point scale (from 0 = never to 3 = six times or more). If participants endorsed “never” on all seven NSSI items, it indicated that the individual had not engaged in NSSI. If participants reported engaging in one or more instances of NSSI, it indicated that the individual had engaged in NSSI in the last 6 months. The detection rate of NSSI was calculated by dividing the number of individuals who reported engaging in one or more instances of NSSI by the total number of individuals. The reliability and validity of this questionnaire among Chinese adolescent were satisfactory [25]. In current study, the Cronbach’s α is 0.81.

#### Depression-anxiety-stress scale (DASS-21)

The Chinese version of DASS-21 developed by Gong et al. [26] was used to asset the three negative emotions of depression, anxiety and stress among adolescents. The scale contains 21 items, and each of the three subscales of depression, anxiety and stress contains 7 items, all of which are scored on a 4-point scale (from 0 = does not match to 3 = always matches), and the scores of each subscale are multiplied by 2, which is the score for that subscale, with the total score ranging from 0 to 42, and the higher the rating, the greater the likelihood that a person will experience that type of negative emotion. The reliability and validity of the Chinese version of DASS-21 among Chinese adolescent were satisfactory [27]. In current study, the Cronbach’s α is 0.92.

### Statistical analysis

This study employed M-plus Version 8.3 to analyze the latent profiles of adolescents’ CERS and determine their categories and distribution. Descriptive statistics, correlation analysis, ANOVA, logistic regression, and *χ*^2^ tests were conducted by IBM SPSS 22.0 to examine the effects of socio-demographic variables on the latent profiles of CERS, as well as explore the relationship among the latent profiles, negative emotions, and NSSI in adolescents.

## Results

### Test of common method bias

The Harman one-factor method was used to test for common method bias prior to data analysis. And the results revealed that there were 9 factors with eigenvalues greater than 1, as well as the variance explained by the first factor was 20.28%, which is far less than the critical value of 40% [28]. Thus, there is no significant common method bias in current study.

### Data description

Table 1 shows the mean (*M*) and standard deviation (*SD*) of CERQ for all samples. Of the 2711 valid samples in this study, 1065 (39.28%) individuals reported engaging in one or more instances of NSSI and were considered to have been involved in NSSI behavior.

**Fig. 1:**
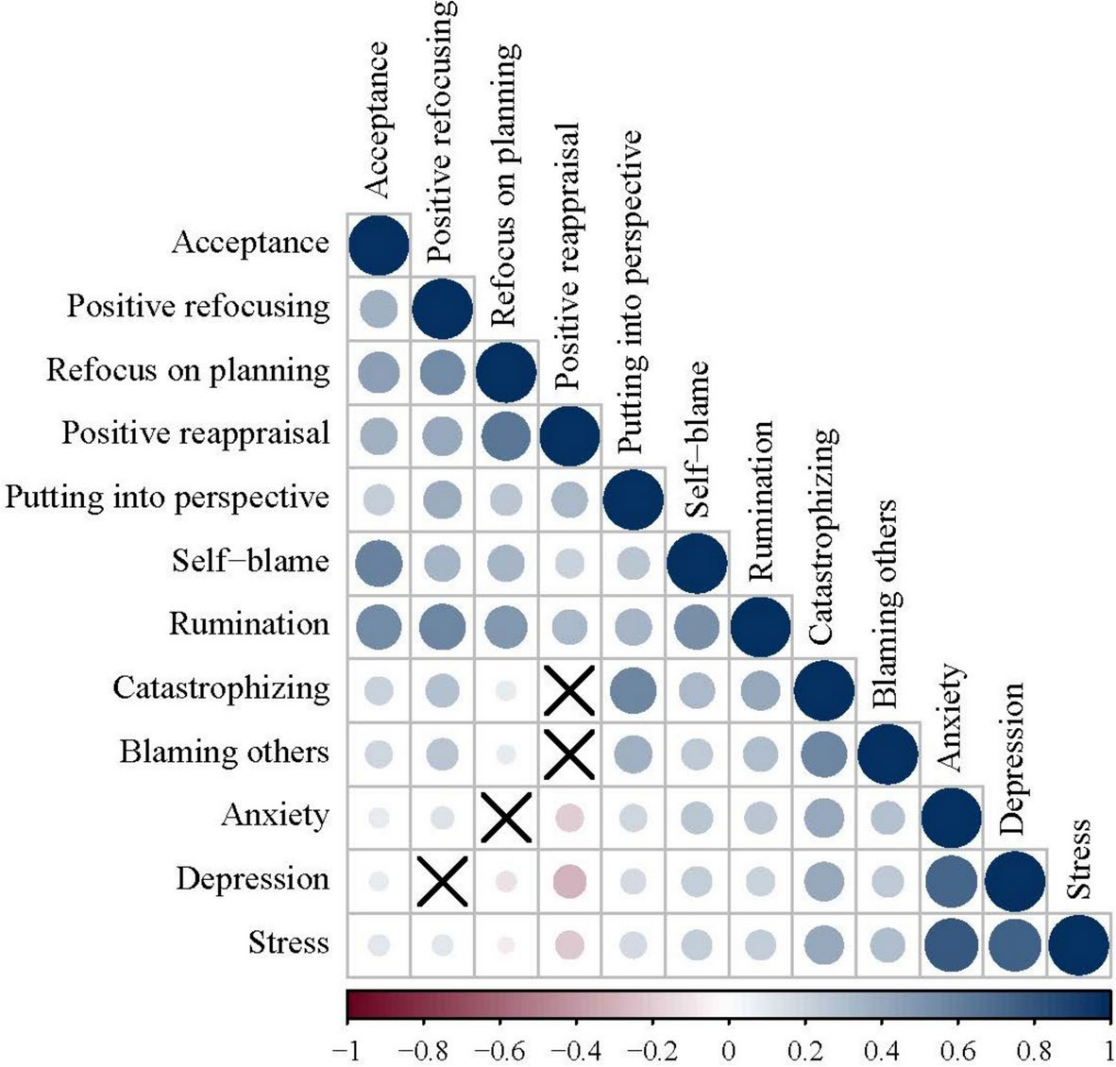
Correlation coefficient of each variable (*n* = 2711)

Figure 1 reveals the correlation between all the variables for all samples. Results showed that all the correlations among variables were at significant levels (*r* = − 0.29 ~ 0.82, *p* < 0.05) except for the association between positive refocusing and depression (*r* = 0.03, *p* = 0.12), refocus on planning and anxiety (*r* = − 0.02, *p* = 0.21), positive reappraisal and catastrophizing (*r* = 0.02, *p* = 0.28) and blaming others (*r* = 0.03, *p* = 0.09).

### Latent profile analysis

Table 2 presents the fit indices for solutions ranging from one to six profiles. The results indicate that the five-profile model is the most optimal solution based on a combination of model fit and parsimony. We evaluated model fit using several indices: Akaike information criterion (AIC), Bayesian information criterion (BIC), adjusted Bayesian information criterion (aBIC), Lo-Mendell-Rubin likelihood ratio test (LMRT), Entropy, and Bootstrap likelihood ratio test (BLRT). AIC, BIC, and aBIC are used to select the most parsimonious model by balancing model fit with model complexity. Lower values of these criteria indicate a better balance between fit and complexity, preventing overfitting.

**Table 2:** Fit statistics for the latent profile analysis (*n* = 2711)

Model	AIC	BIC	aBIC	Entropy	p-value for LMRT	p-value for BLRT
C1	68,626.76	68,733.05	68,675.86	1.00	–	–
C2	64,515.23	64,680.57	64,591.60	0.76	< 0.01	< 0.01
C3	62,689.40	62,913.79	62,793.05	0.83	< 0.05	< 0.01
C4	61,320.08	61,603.53	61,451.01	0.83	< 0.01	< 0.01
C5	60,588.59	60,931.08	60,746.80	0.82	< 0.01	< 0.01
C6	60,068.11	60,469.66	60,253.60	0.81	0.39	< 0.01

*AIC* Akaike information criterion, *BIC* Bayesian information criterion, *aBIC* adjusted- Bayesian information criterion, *LMRT* Lo-Mendell-Rubin likelihood ratio test, *BLRT* bootstrap likelihood ratio test

LMRT and BLRT were used to compare the goodness-of-fit between models, with *P* < 0.05 indicating that a model with k profiles is significantly better than a model with k-1 profiles. The five-profile model shows lower AIC, BIC, and aBIC values compared to the four-profile model and also demonstrates a superior entropy value compared to the six-profile model, suggesting clearer classification.

Furthermore, the additional class in the six-profile model may result from splits driven by extreme responses from a small subset of participants, particularly on maladaptive CERS dimensions such as ‘Self-blame’ and ‘Rumination’. These extreme responses likely distort the classification process, introducing artificial splits that lack meaningful theoretical differentiation. This interpretation is further supported by the observation that these additional classes lack clear conceptual boundaries and exhibit substantial overlap with the classes identified in the five-profile model (Details of the six-profile model are provided in Table S3).

In conclusion, the five-profile model of CERS items provides not only the best fit according to statistical indices but also the most interpretable and theoretically coherent classification solution.

**Fig. 2:**
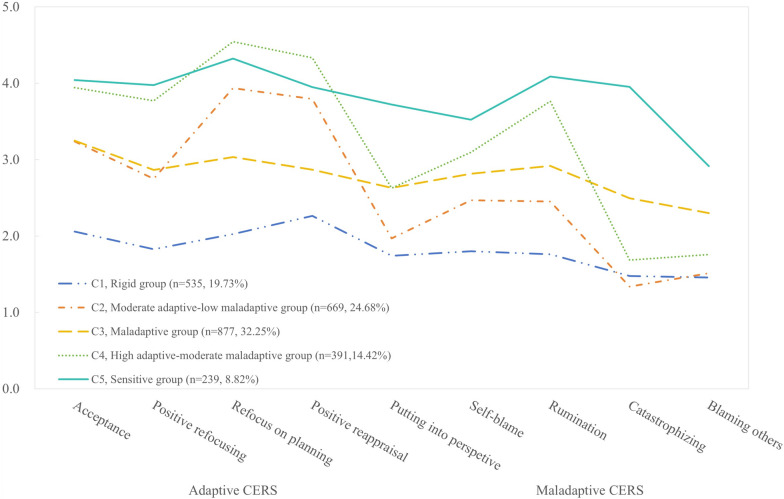
Estimated mean values for latent profiles (*n* = 2711)

Figure 2 shows the scores of the five latent profiles of adolescents’ CERS. The naming of the five latent profiles was based on the differences in their scores on adaptive and maladaptive CERS. The five profiles comprised 19.73%, 24.68%, 32.25%, 14.42% and 8.82% of adolescents.

Table 3 shows the results of the one-way ANOVA, indicating statistically significant differences between the various latent profiles on adaptive and maladaptive CERS. Post hoc tests revealed that adolescents belonging to C1 had the lowest scores on both adaptive and maladaptive CERS. This suggests that the CERS of this group of adolescents is more rigid, and they can be classified as the ‘Rigid group’; Adolescents belonging to C2 have scores closer to the mean for adaptive CERS and lower scores for maladaptive CERS, and they can be classified as the ‘Moderate adaptive-low maladaptive group’; Adolescents belonging to C3 have higher scores than C1 for adaptive CERS and lower scores than C5 for maladaptive CERS, suggesting that the CERS of this group is more markedly maladaptive, which can be referred to as the ‘Maladaptive group’; C4 adolescents exhibit higher scores in adaptive CERS, while their maladaptive CERS scores are close to the average. This can be classified as a ‘High adaptive-moderate maladaptive group’; C5 adolescents exhibit the highest scores in both adaptive and maladaptive CERS, suggesting that the CERS of this type of adolescents is more sensitive, which can be classified as ‘Sensitive group’.

**Table 3:** Differences in CERS across the five latent profiles

	Adaptive CERS	Maladaptive CERS
Total (*n* = 2711)	15.11 ± 3.80	9.37 ± 2.73
C1 (*n* = 535)	9.87 ± 2.27	6.47 ± 1.50
C2 (*n* = 669)	15.69 ± 1.92	7.69 ± 1.29
C3 (*n* = 877)	14.64 ± 1.89	10.58 ± 1.46
C4 (*n* = 391)	19.33 ± 1.82	10.35 ± 1.35
C5 (*n* = 239)	20.05 ± 2.65	14.51 ± 2.07
*F*	1669.18**	1642.55**
*Comparison(LSD)*	C1 < C3 < C2 < C4 < C5	C1 < C2 < C4 < C3 < C5

Total sample is not included as a group in ANOVA

C1 = ‘Rigid group’, C2 = ‘Moderate adaptive-low maladaptive group’, C3 = ‘Maladaptive group’, C4 = ‘High adaptive-moderate maladaptive group’, C5 = ‘Sensitive group’; LSD: Least Significant Difference

**p < 0.01

### Association between demographic characteristics and the latent profiles

Taking the ‘Sensitive group’(C5) as reference, the ‘Rigid group’ (C1), ‘Moderate adaptive-low maladaptive group’(C2), ‘Maladaptive group’(C3) and the ‘High adaptive-moderate maladaptive group’ (C4) were compared. Odds ratio (OR) results showed that the latent profile of CERS was influenced by demographic factors such as age, gender, whether it was an only child, and whether it has left-behind experience(*P* < 0.05). Results of multiple logistic regression analysis indicate that age and gender were significant predictors of the latent profile of adolescents’ CERS (Table 4). Specifically, for each one-year increase in age, adolescents were 0.812 times more likely to belong to the C2 than to the C5 (*OR* = 0.812, 95% *CI* = 0.66–0.99, *p* < 0.05). The odds ratio (OR) for males belonging to the C2 compared to the C5 was 0.698 (95%*CI* = 0.52–0.94, *p* < 0.05), indicating a lower likelihood than females.

**Table 4:** Multinomial regression analysis of different classes with C5 as reference

		C1		C2		C3		C4	
		OR	95%***CI***	OR	95%***CI***	OR	95%***CI***	OR	95%***CI***
Age		1.105	0.90–1.36	0.812*	0.66–0.99	1.034	0.85–1.26	0.859	0.69–1.07
Gender	Male	0.933	0.69–1.27	0.698*	0.52–0.94	0.870	0.65–1.16	0.932	0.67–1.29
	Female								
OC	Yes	1.103	0.69–1.76	1.301	0.83–2.04	1.243	0.80–1.93	1.184	0.73–1.93
	No								
LBE	Yes	0.862	0.59–1.25	0.758	0.52–1.10	1.112	0.79–1.57	0.852	0.57–1.27
	No								

Results are shown as odds ratios with 95% confidence intervals

OC: Only child in the family, LBE: Left-behind experience

C1 = ‘Rigid group’, C2 = ‘Moderate adaptive-low maladaptive group’, C3 = ‘Maladaptive group’, C4 = ‘High adaptive-moderate maladaptive group’, C5 = ‘Sensitive group’

*p < 0.05

### Association between the negative emotions, NSSI and the latent profiles

The results of the ANOVA (Table 5) indicated statistically significant differences (*p* < 0.01) in the scores of three negative emotions (anxiety, depression, and stress) among adolescents with different latent profiles of CERS. Further multiple comparisons revealed that the trend of differences in anxiety and stress scores among adolescents across the five latent profiles was the same, with the C2 having significantly lower scores than the C1, and that there was no significant difference between the scores of these two groups and the scores of the other three groups; among the other three groups, the C4 had the lowest anxiety and stress scores, the C3 had higher scores, and the C5 had the highest scores. Meanwhile, depression scores varied significantly between the five groups. The most C5 was followed by the C3, then the C1 in third place, the C4 in fourth, and finally the C2 in last.

**Table 5:** Differences in negative emotions across the five latent profiles

	Anxiety	Depression	Stress	NSSI
	***M*** ± ***SD***	***M*** ± ***SD***	***M*** ± ***SD***	***n*** (***%***)
Total (*n* = 2711)	7.63 ± 6.98	6.31 ± 7.08	9.66 ± 8.04	1065(39.28)
C1 (*n* = 535)	5.91 ± 5.74	5.48 ± 6.33	8.10 ± 7.05	186(34.77)
C2 (*n* = 669)	4.74 ± 4.87	3.09 ± 4.00	6.13 ± 5.92	142(21.23)
C3 (*n* = 877)	9.93 ± 7.05	8.81 ± 7.59	12.38 ± 7.85	472(53.82)
C4 (*n* = 391)	6.16 ± 6,11	4.11 ± 5.33	7.82 ± 7.15	116(29.67)
C5 (*n* = 239)	13.57 ± 9.02	11.60 ± 9.37	16.01 ± 10.09	149(62.34)
*F*/*c*^2^	128.67**	126.08**	123.76**	242.17**
Comparison(*LSD*/*Bonferroni*)	C2 < C1; C4 < C3 < C5	C2 < C4 < C1 < C3 < C5	C2 < C1; C4 < C3 < C5	C2 < C1; C4 < C3, C5

Total sample not included as a group in ANOVA/ c^2^ test

C1 = ‘Rigid group’, C2 = ‘Moderate adaptive-low maladaptive group’, C3 = ‘Maladaptive group’, C4 = ‘High adaptive-moderate maladaptive group’, C5 = ‘Sensitive group’

**p < 0.01, *p < 0.05

The *c*^2^ test results (Table 5) indicated statistically significant differences (*p* < 0.01) in the detection rates of NSSI among adolescents with different latent profiles of CERS. The detection rate of NSSI was significantly lower in the C2 than in the C1. Additionally, it was also significantly lower in the C4 than in the C3 and C5.

## Discussion

### latent profiles of CERS among Chinese junior high school students

The current study revealed that Chinese junior high school students’ CERS can be classified into five latent profiles. The largest group was the ‘Maladaptive group’ (32.25%), followed by the ‘Moderate adaptive-low maladaptive group’ (24.68%), ‘Rigid group’ (19.73%), and ‘High adaptive-moderate maladaptive group’ (14.42%). The remaining group was the ‘Sensitive group’ (C5, 8.82%).

Both this study and Chen’s (2018) et al. study have found that, in contrast to Dutch individuals, the proportion of ‘Adaptive Regulators’ characterized by a clear adaptive tendency and low maladaptive tendency may be small among Chinese adolescents, making accurate identification difficult [21, 22]. This discrepancy is possibly due to the influence of different social cultures on the utilization of CERS by individuals in various countries. And this conclusion needs to be verified by national studies with large sample size.

Furthermore, this study builds upon the work of Chen et al. [22]. The findings reveal that the ‘medium reaction profile’ of CERS in Chinese adolescents can be categorized into a ‘High adaptive-moderate maladaptive group’ and a ‘Moderate adaptive-low maladaptive group. This indicates that the ‘medium reaction profile’ may not simply represent average levels in both adaptive and maladaptive CERS, but rather demonstrate average levels in one aspect while exhibiting relatively high or low features in the other. Once again, these results underscore the highly diverse and individualized nature of CERS.

### Demographic characteristics of latent profiles of CERS

The study results indicate that age and gender were predictive of latent categories of CERS among Chinese junior high school students. For each 1-year increase in age, the proportion of junior high school students in the ‘Moderate adaptive-low maladaptive group’ was lower than in the ‘Sensitive group’. This finding is consistent with previous research and suggests that adolescence is an important stage in the development of an individual’s affective functioning, during which individuals continue to learn and use CERS to construct their own emotion regulation functioning [29, 30]. It is suggested that teachers and parents should focus on developing the affective functioning of middle school students, guiding them to master affective CERS, reduce maladaptive CERS, and develop stable and positive cognitive emotion regulation to cope with the effects of negative emotions on the individual’s physical and mental health.

This study also found that males were less distributed in the ‘Moderate adaptive-low maladaptive group’ than in the ‘Sensitive group’. The evidence suggests that male tend to use a more active CERS (both active in adaptive and maladaptive CERS), while female tend to use a more conservative strategy. This finding is consistent with previous research indicating that gender differences in CERS reflect societal expectations of gender roles [31]. Men are generally expected to be more adept at coping with stress and challenges and therefore may need to use more active CERS to better cope with emotional stress; whereas socio-cultural demands on women to cope with stress are less stringent than on men, as evidenced by the fact that women are allowed to have more emotional relief and to withdraw from stressful situations, and therefore women may take a relatively conservative CERS [32, 33]. However, this study suggests that the more frequent use of CERS by men does not indicate better mental health, rather the ‘Sensitive group’ had the highest levels of anxiety, depression, distress and NSSI behaviors. A possible reason for this is that overuse of CERS is associated with burnout of physical and mental resources, which makes men more likely to exhibit relatively severe emotional-behavioral problems in the midst of constant stress.

### CERS profiles in junior high school students in relation to negative emotions and NSSI

The relationship between potential CERS profiles and negative emotions and NSSI among Chinese junior high school students is revealed for the first time in this study. The results of this study showed that the ‘Sensitive group’ had the highest anxiety, depression and stress scores and NSSI detection rates, suggesting that the emotional-behavioral problems experienced by junior high school students in the ‘Sensitive group’ were most severe, which is similar to the findings of a study in a sample of nurses, indicating that very frequent use of CERS may not imply that individuals have benign emotion regulation [34]. Instead, this sensitivity to cognitive emotion regulation may trigger more psychosomatic distress. One possible explanation is that the high frequency of CERS use may reflect that individuals in this group are more sensitive to external stimuli and internal mood changes, and thus more vulnerable to negative emotions.

In addition, the present study found that anxiety, depression and stressful emotions and NSSI detection rates in the ‘Maladaptive group’ were at high levels, which is consistent with the findings of previous studies, suggesting that maladaptive CERS is an important risk factor for emotional-behavioral problems in adolescents [35, 36]. This suggests that clinical psychological interventions should focus on improving maladaptive CERS in adolescents in order to mitigate its negative impact on mental health outcomes.

The results of the present study also showed that the ‘Rigid group’ and the ‘High adaptive-moderate maladaptive group’ had relatively mild emotional-behavioral problems. The ‘Moderately adaptive-low maladaptive group’ had the mildest emotional-behavioral problem status. The common feature of these three groups of adolescents is a low level of maladaptive CERS. This suggests that lower levels of maladaptive CERS are protective factors for adolescents’ negative emotions and NSSI problems [37]. The possible reason for this is that adolescents with lower levels of maladaptive CERS are less likely to fall into negative emotional cycles, such as rumination and negative thinking, and are able to proactively seek social support and help, thereby better coping with their emotional distress and reducing the likelihood of transmitting their emotional pain through NSSI.

The above findings indicate that strategies must be tailored and implemented according to the specific characteristics of adolescent CERS when preventing and intervening in adolescent negative emotions and NSSI. While all latent profiles of CERS are important for adolescents’ anxiety, depression, stressful emotions and NSSI, mental health practitioners should pay particular attention to the ‘Sensitive group’ of CERS. This group is at high risk of emotional-behavioral problems and requires early identification and intervention to help them cope with emotional distress, and prevent further deterioration of mental health problems.

## Limitations and implications

The present study is subject to certain limitations. Firstly, the self-report method was employed to assess CERS in this study, which may have introduced a degree of information bias. Future studies may therefore benefit from utilizing a more comprehensive approach, such as behavioral experiments, to measure CERS. Secondly, this study identified differences in the latent profiles of CERS between Chinese and Dutch adolescents. However, it did not examine these differences in depth. The reasons for such differences remain unclear, and future studies may explore the reasons from a cross-cultural perspective. At the same time, due to the limited number of relevant studies, the results obtained in this research could not be adequately compared with similar findings from China and the Netherlands. Future research can address this gap and enhance the credibility of cross-cultural psychological studies. Finally, although this study examined high-risk groups for negative emotions and NSSI in Chinese junior high school students, the cross-sectional study design did not allow for the determination of the relationship between the latent profiles of CERS and negative emotions and NSSI. Future research could employ longitudinal studies to further substantiate the impact of CERS on adolescents’ emotional-behavioral problems.

## Conclusion

In general, the differences in anxiety, depression, stress, and NSSI indicators among Chinese junior high school students with different CERS profiles were statistically significant. The ‘Sensitive group’ exhibited the most severe emotional-behavioral problems, the ‘Maladaptive group’ was more severe, the ‘Rigid group’ and the ‘High Adaptive-Moderate Maladaptive group’ exhibited less severe problems, and the ‘Moderate Adaptive-Low Maladaptive group’ exhibited the mildest. The findings of this study indicate that mental health practitioners should prioritize the assessment and intervention of CERS in junior high school students. This is crucial to reduce the incidence of emotional-behavioral problems.

## Supplementary Information

Supplementary Material 1.

Supplementary Material 2.

Supplementary Material 3.

## Data Availability

The datasets during the current study are not publicly available but are available from the corresponding author on a reasonable request.

## Abbreviations

CERS: Cognitive Emotion Regulation Strategies
NSSI: Non-suicidal self-injury
OC: Only child in the family
LBE: Left-behind experience
AIC: Akaike information criterion
BIC: Bayesian information criterion
aBIC: Adjusted-Bayesian information criterion
LMRT: Lo-Mendell-Rubin likelihood ratio test
BLRT: Bootstrap likelihood ratio test
C1: Rigid group
C2: Moderate adaptive-low maladaptive group
C3: Maladaptive group
C4: High adaptive-moderate maladaptive group
C5: Sensitive group
