# Supplementary material for: A latent profile analysis of cognitive emotion regulation strategies in relation to negative emotions and NSSI among Chinese junior high school students
Source: Child Adolesc Psychiatry Ment Health. 2024 Dec 4;18:155. doi: 10.1186/s13034-024-00838-5 (PMC11619670; doi:10.1186/s13034-024-00838-5)
Supplement: Supplementary file 1 — Supplementary Material 1. [file 13034_2024_838_MOESM1_ESM.pdf]

Table S1 *Socio- demographic questionnaire*

1.Your gender

a. Male

b. Female

2.Your age_______

3.Have you ever been away from your parents and in the care of others before 16 years old?

a. Yes

b. No

4.Are you an only child in your family?

a. Yes

b. No

5. Have you ever suffered from a mental or psychological illness?

a. Yes

b. No
