# Supplementary material for: A latent profile analysis of cognitive emotion regulation strategies in relation to negative emotions and NSSI among Chinese junior high school students
Source: Child Adolesc Psychiatry Ment Health. 2024 Dec 4;18:155. doi: 10.1186/s13034-024-00838-5 (PMC11619670; doi:10.1186/s13034-024-00838-5)
Supplement: Supplementary file 2 — Supplementary Material 2. [file 13034_2024_838_MOESM2_ESM.pdf]

Table S2. Correlation coefficients between variables.

	Acceptance	Positive refocusing	Refocus on planning	Positive reappraisal	Putting into perspective	Self-blame	Rumination	Catastrophizing	Blaming others	Anxiety	Depression
Positive refocusing	0.371**										
Refocus on planning	0.453**	0.543**									
Positive reappraisal	0.364**	0.411**	0.645**								
Putting into perspective	0.240**	0.381**	0.261**	0.335**							
Self-blame	0.585**	0.345**	0.354**	0.216**	0.272**						
Rumination	0.540**	0.574**	0.481**	0.323**	0.353**	0.539**					
Catastrophizing	0.204**	0.290**	0.093**	0.021	0.573**	0.332**	0.407**				
Blaming others	0.188**	0.265**	0.081**	0.033	0.372**	0.252**	0.312**	0.563**			
Anxiety	0.100**	0.129**	-0.024	-0.190**	0.188**	0.261**	0.260**	0.407**	0.291**		
Depression	0.085**	0.030	-0.100**	-0.287**	0.160**	0.237**	0.212**	0.407**	0.258**	0.729**	
Stress	0.108**	0.106**	-0.061**	-0.207**	0.177**	0.240**	0.236**	0.403**	0.307**	0.783**	0.747**
