# Supplementary material for: A latent profile analysis of cognitive emotion regulation strategies in relation to negative emotions and NSSI among Chinese junior high school students
Source: Child Adolesc Psychiatry Ment Health. 2024 Dec 4;18:155. doi: 10.1186/s13034-024-00838-5 (PMC11619670; doi:10.1186/s13034-024-00838-5)
Supplement: Supplementary file 3 — Supplementary Material 3. [file 13034_2024_838_MOESM3_ESM.pdf]

Table S3 Differences in CERS across the six latent profiles.

	Acceptance	Positive refocusing	Refocus on planning	Positive reappraisal	Putting into perspective	Self-blame	Rumination	Catastrophizing	Blaming others
C1 (n=667)	2.64	2.27	2.53	2.56	1.99	2.23	2.20	1.68	1.68
C2 (n=160)	1.45	1.31	1.42	1.86	1.42	1.36	1.29	1.25	1.19
C3 (n=634)	3.31	2.81	4.12	3.98	2.03	2.51	2.52	1.36	1.54
C4 (n=738)	3.36	3.01	3.19	2.98	2.76	2.91	3.09	2.68	2.41
C5 (n=338)	3.98	3.89	4.59	4.36	2.70	3.14	3.85	1.73	1.75
C6 (n=174)	4.13	4.06	4.44	4.10	3.86	3.62	4.18	4.09	3.07
